# PVP-functionalized Dy_2_O_3_/Pr_2_O_3_ nanocomposites: a multifunctional platform for photoluminescence, WLEDs, latent fingerprinting, and anti-counterfeiting applications

**DOI:** 10.1039/d5ra06661a

**Published:** 2025-09-26

**Authors:** Kartik Gopal, Sunitha D. V.

**Affiliations:** a Department of Physics, School of Applied Sciences, REVA University Bengaluru-560064 Karnataka India sunithaprasad8@gmail.com; b Center for Advance Materials and Research in Physics, REVA Research Center, REVA University Bengaluru-560064 Karnataka India

## Abstract

Luminescent nanomaterials that combine high optical performance with multifunctional utility are essential for next-generation optoelectronic and forensic applications. However, achieving simultaneous enhancement of photoluminescence efficiency and practical applicability remains a challenge. Here, we report the synthesis of PVP-functionalized Dy_2_O_3_/Pr_2_O_3_ nanocomposites *via* a solution combustion route, where surface passivation with 9 wt% polyvinylpyrrolidone (PVP) effectively reduced crystallite size and improved dispersion. Structural and spectroscopic analyses confirmed strong polymer–oxide interactions, leading to defect passivation and enhanced luminescence. Photoluminescence studies demonstrated intense Dy^3+^–Pr^3+^ emissions with a prolonged decay lifetime (5.2 ns) and a high quantum yield (92.16%). Chromaticity analysis indicated warm white light emission (CCT ≈ 5826 K, CRI ≈ 92), underscoring their suitability for high-quality WLEDs. Beyond lighting, the nanocomposites enabled clear visualization of latent fingerprints under UV illumination, revealing level 1–3 ridge details, and served as a covert UV-responsive ink for anti-counterfeiting applications. These results position PVP-coated Dy_2_O_3_/Pr_2_O_3_ nanocomposites as a versatile platform bridging solid-state lighting, forensics, and security technologies.

## Abstract 2

PVP-coated Dy_2_O_3_/Pr_2_O_3_ nanocomposites exhibit intense warm-white luminescence and enable clear latent fingerprint visualization, demonstrating multifunctional potential for WLEDs, forensics, and anti-counterfeiting applications.

## Graphical abstract

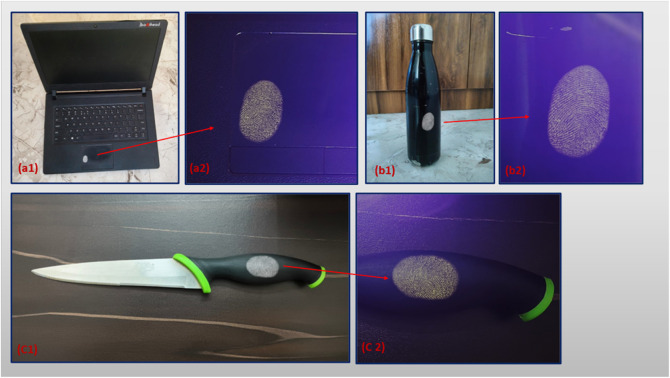

## 1. Introduction

Rare-earth oxide (REO) nanomaterials continue to play a pivotal role in the advancement of optoelectronic and security-related technologies due to their superior luminescent properties, thermal robustness, and unique 4f-electron configurations.^1–4^ Among these, dysprosium oxide (Dy_2_O_3_) is widely recognized for its strong blue (∼480 nm) and yellow (∼575 nm) photoluminescence, thermal resilience, and chemical inertness, making it an effective phosphor for applications such as white light-emitting diodes (WLEDs),^5–7^ latent fingerprint (LFP) visualization,^8,9^ and anti-counterfeiting systems.^10–12^ Praseodymium oxide (Pr_2_O_3_), another lanthanide sesquioxide, offers broad emission bands from green to red under UV excitation and demonstrates excellent red-green chromatic tunability through intra-4f transitions.^13,14^ However, when individually used, both the materials has its own limitations. Dy_2_O_3_ suffers from insufficient red emission for high color rendering in WLEDs, and Pr_2_O_3_ alone lacks sufficient intensity in the yellow region.

To overcome these spectral limitations and improve multifunctional performance, we developed a Dy_2_O_3_/Pr_2_O_3_ nanocomposite, which leverages the cooperative energy transfer between Dy^3+^ and Pr^3+^ ions. This compositional integration enables more complete spectral coverage and optimized emission balance under UV excitation, which is particularly beneficial for single-material WLED phosphor design and high-contrast luminescent tagging. The nanocomposite approach also enhances crystallographic stability and reduces the concentration quenching commonly observed in highly doped single-ion systems.^15–18^ Compared to conventional host-doped phosphors, binary oxide nanocomposites offer an alternative path to achieve multi-emission systems with reduced synthesis complexity and improved material yield.

Current commercial phosphors used in WLEDs include Ce^3+^ doped yttrium aluminum garnet (YAG:Ce^3+^), which offers high quantum efficiency but suffers from high correlated color temperature (CCT), poor red emission, and color rendering index (CRI) limitations.^19–22^ Other garnet systems, such as (Gd,Y)_3_Al_5_O_12_:Ce^3+^,^23,24^ and (Lu,Gd)AG:Ce^3+^,^25^ have been explored for improved spectral control, but these materials suffer from limited red emission. Silicate-based phosphors such as Sr_2_SiO_5_:Eu^2+^,^26,27^ and BaSi_2_O_5_:Eu^2+^,^28,29^ provide broader emissions but exhibit structural degradation under long-term thermal exposure. Aluminate phosphors like BaMgAl_10_O_17_:Eu^2+^,^30^ and SrAl_2_O_4_:Eu^2+^,^31^ offer excellent blue-green emission but suffer from poor emission longevity and fast decay. Nitride-based phosphors CaAlSiN_3_:Eu^2+^,^32^ demonstrate strong red emission and thermal stability but involve complex and costly synthesis procedures. Perovskite phosphors CsPbBr_3_ ^33–35^ and quantum dots CdSe,^36,37^ InP-based,^38,39^ show high luminescence and tunability but face issues related to moisture sensitivity, thermal instability, and toxicity, limiting their practical scalability.

In this context, rare-earth oxide (REO) nanocomposites offer a compelling alternative due to their stability, broad excitation/emission profiles, and simpler synthetic processing. Yet, despite the strong potential of Dy_2_O_3_ and Pr_2_O_3_ individually, there is limited research exploring their integrated behavior within a co-oxide nanocomposite framework. The cooperative luminescence dynamics between Dy^3+^ and Pr^3+^ remain underexplored, particularly for multifunctional applications requiring both efficient light emission and substrate-level interaction, as in LFP and anti-counterfeiting materials. Additionally, REO nanoparticles are often limited by surface defects, agglomeration, and oxygen vacancies, which degrade luminescence efficiency and shorten emission lifetimes.^40,41^

To address these shortcomings, surface passivation using polyvinylpyrrolidone (PVP) was employed in this work. PVP is a biocompatible, non-ionic polymer widely used for stabilizing nanoparticles due to its ability to reduce surface defects, inhibit aggregation, and provide hydrophilic functionality. These properties are especially advantageous for dispersing luminescent materials into inks or fingerprinting powders, as well as enhancing quantum efficiency by passivating non-radiative surface states. While previous studies have investigated PVP-coated Dy_2_O_3_ nanoparticles,^42^ no systematic work has been reported on PVP-coated Dy_2_O_3_/Pr_2_O_3_ nanocomposites, particularly in the context of WLEDs, anti-counterfeiting, and forensic imaging applications.

In the present study, we synthesized a Dy_2_O_3_/Pr_2_O_3_ nanocomposite *via* solution combustion synthesis a rapid, energy-efficient method known for producing highly crystalline, porous nanocomposite and cost effective. To improve the optical response and application performance, the nanocomposite was subsequently coated with PVP *via* solution-phase stirring and drying. The structural, morphological, and photoluminescent characteristics of the synthesized material were systematically analyzed, and its practical utility was assessed across three critical domains: white light emission, high-contrast latent fingerprint visualization under UV excitation, and anti-counterfeiting functionality. This work establishes a new multifunctional luminescent material platform and provides a scalable strategy for designing rare-earth-based nanophosphors with enhanced optical and practical performance.

## 2. Experimental methods

### 2.1. Materials

99.99% pure dysprosium(iii) nitrate hexahydrate (Dy(NO_3_)_3_·6H_2_O), urea (NH_2_CO_2_NH), praseodymium(iii) nitrate hexahydrate (Pr(NO_3_)_3_·6H_2_O), polyvinylpyrrolidone (C_6_H_9_NO-PVP K-30) and ethanol (C_2_H_5_OH) were procured from Sigma-Aldrich. The raw materials procured are of analytical grade were used directly for material preparation.

### 2.2. Experimental procedure

For the synthesis of the Dy_2_O_3_/Pr_2_O_3_ nanocomposite, stoichiometric amounts of dysprosium(iii) nitrate hexahydrate (Dy(NO_3_)_3_·6H_2_O, 1.75255 g) and praseodymium(iii) nitrate hexahydrate (Pr(NO_3_)_3_·6H_2_O, 1.75255 g) was taken in 1 : 1 molar ratio by weighing precisely and dissolved together in a 20 mL of deionized water. Urea (1.5375 g) was used as the fuel and added to the solution. The resulting mixture was then stirred at 350 rpm for 30 min to ensure homogeneity. The homogeneous precursor solution was then transferred into an alumina crucible and placed in a preheated muffle furnace maintained at 500 °C. Within 5 min, a self-propagating combustion reaction occurred, producing a porous solid mass. The foamy product was scraped out, grounded using an agate mortar and pestle, and then calcined at 900 °C for 4 h in air atmosphere to improve its crystallinity and phase purity.

For surface coating, 0.5 g of the prepared Dy_2_O_3_/Pr_2_O_3_ nanocomposite powder was mixed with 0.045 g of polyvinylpyrrolidone (PVP, 9 wt%) in 20 mL of absolute ethanol. The mixture was stirred at 350 rpm for 24 h at room temperature to promote uniform coating. Following the adsorption process, the suspension was dried at 50 °C for 24 h to evaporate the solvent and yield dry, PVP-coated Dy_2_O_3_/Pr_2_O_3_ nanocomposite. For preparing luminescent security ink, 100 mg of the dried PVP-coated Dy_2_O_3_/Pr_2_O_3_ nanocomposite powder was redispersed in 2 mL of absolute ethanol containing 2 wt% PVP as a stabilizer. The mixture was ultrasonicated for 30 min to obtain a homogeneous suspension with good stability and wettability. This dispersion was used directly as a functional ink for dip-pen inscription on various real-world substrates, as demonstrated in the anti-counterfeiting experiments.

### 2.3. Characterization methods

The structural and phase analysis of the synthesized samples were conducted using a Rigaku Smart Lab Powder X-ray Diffractometer (PXRD) with CuKα radiation (*λ* = 1.541 Å) over the 2*θ* range of 20°–80° at a scan rate of 2° min^−1^. Fourier Transform Infrared (FTIR) spectroscopy were performed on a Bruker Alpha II FTIR spectrophotometer, operating in the mid-infrared region (4000–400 cm^−1^) with a resolution of 0.9 cm^−1^. Both transmission and attenuated total reflectance (ATR) modes were utilized with a KBr beam splitter. The surface morphology and elemental composition were analyzed using a VEGA3 TESCAN Field Emission Scanning Electron Microscope (FESEM), which operates at 0.2–30 kV and includes an Energy Dispersive X-ray Spectroscopy (EDX) system for elemental analysis. Optical properties were examined using a Thermo Scientific UV-Visible spectrophotometer, which recorded absorbance in 200–800 nm range, utilizing a 15 W fluorescent UV lamp as the light source. High-Resolution Transmission Electron Microscopy (HRTEM) characterization were performed using a Thermofisher Talos F200S G2 instrument. This state of the art system operates at 200 kV and is equipped with a Field Emission Gun (FEG), a CMOS camera with a resolution of 4k × 4k, and an in-column Energy Dispersive X-ray Spectroscopy (EDS) detector. This advanced characterization facilitated detailed insights into the structural, morphological, and elemental properties of the samples. In photoluminescence (PL) studies, emission, excitation, including lifetime and quantum yield measurements, were recorded using an FLS1000 Edinburgh Instrument. The excitation sources comprised a 450 W Xenon arc lamp (250–900 nm) and a 200 mW He–Cd laser (325 nm). Time-resolved measurements were carried out using microsecond flash lamps and pulsed lasers or LEDs at specific wavelengths, and emissions were detected with visible PMT 900 and NIR PMT 1700 detectors. For imaging latent fingerprint impressions and anti-counterfeiting, a Canon 80D DSLR camera was employed for high-resolution visualization.

## 3. Result and discussion

### 3.1. Phase analysis

**Fig. 1:**
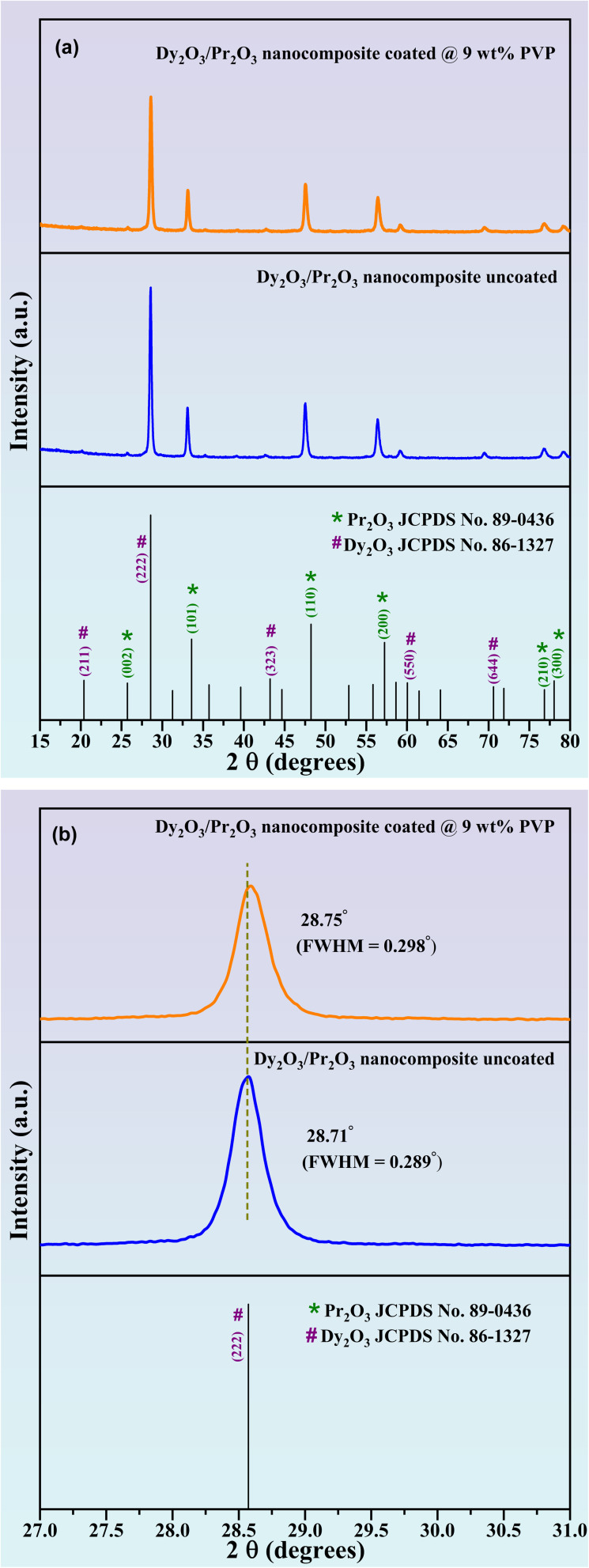
PXRD patterns of (a) uncoated and 9 wt% PVP coated Dy_2_O_3_/Pr_2_O_3_ nanocomposite. (b) Enlarged view of PXRD patterns in the 2*θ* range 27–31°.

The phase purity and crystallographic structure of the synthesized Dy_2_O_3_/Pr_2_O_3_ nanocomposites were examined using X-ray diffraction (XRD), as illustrated in Fig. 1(a and b). The diffraction patterns exhibit well-defined and sharp peaks, indicating high crystallinity in both uncoated and PVP-coated samples. All prominent reflections in the uncoated nanocomposite can be indexed to the cubic phase of Dy_2_O_3_ (JCPDS No. 86-1327, marked with #)^43^ and Pr_2_O_3_ (JCPDS No. 89-0436, marked with *), confirming the successful formation of a Dy_2_O_3_/Pr_2_O_3_ nanocomposite with no observable secondary or impurity phases. The distinct peaks corresponding to (222), (440), and (622) planes of Dy_2_O_3_, along with the (002), (101), and (110) planes of Pr_2_O_3_, further validate the co-existence of both phases within the nanocomposite.

A comparison of the coated and uncoated samples reveals that the overall peak positions remain largely unchanged, indicating that PVP surface functionalization does not alter the fundamental crystal structure. However, a noticeable broadening of diffraction peaks, particularly the (222) reflection of Dy_2_O_3_, is observed in the PVP-coated sample, as shown in the magnified region of Fig. 1(b). This peak slightly shift and broadened due to its reduction in crystallite size and increased microstrain, likely induced by surface passivation and steric hindrance effects from the polymeric coating.^42^

The average crystallite size was estimated using the Debye–Scherrer equation,^44,45^1
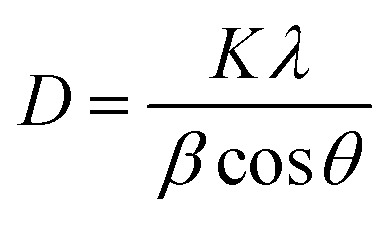
where *D* is the crystallite size, *K* is the shape factor (0.9), *λ* is the X-ray wavelength (1.5406 Å), *β* is the full width at half maximum (FWHM) in radians, and *θ* is the Bragg's angle. The uncoated Dy_2_O_3_/Pr_2_O_3_ nanocomposite exhibited a peak at 2*θ* = 28.71° with FWHM = 0.289°, corresponding to an average crystallite size of ∼22 nm. For the PVP-coated sample, the peak was slightly shifted to 2*θ* = 28.75° with FWHM = 0.298°, yielding a reduced crystallite size of ∼16 nm. The calculated parameters are summarized in Table 1. This ∼7 nm size reduction is consistent with the peak broadening trend and confirms that PVP effectively suppresses particle growth by introducing steric hindrance and microstrain during post-synthesis processing. The smaller crystallite size also enhances the surface-to-volume ratio, which improve optical properties and practical performance in applications such as WLEDs, fingerprint visualization, and anti-counterfeiting inks.

**Table 1:** Estimated crystallite size and band gap values of pure and 9 wt% PVP coated Dy_2_O_3_/Pr_2_O_3_ nanocomposite

Samples	Average crystallite Size(nm) Debye–Scherrer's method (*d*)	Band gap (eV)
Dy_2_O_3_/Pr_2_O_3_ nanocomposite	22	4.90
Dy_2_O_3_/Pr_2_O_3_ nanocomposite coated with 9 wt% PVP	16	4.95

**Fig. 2:**
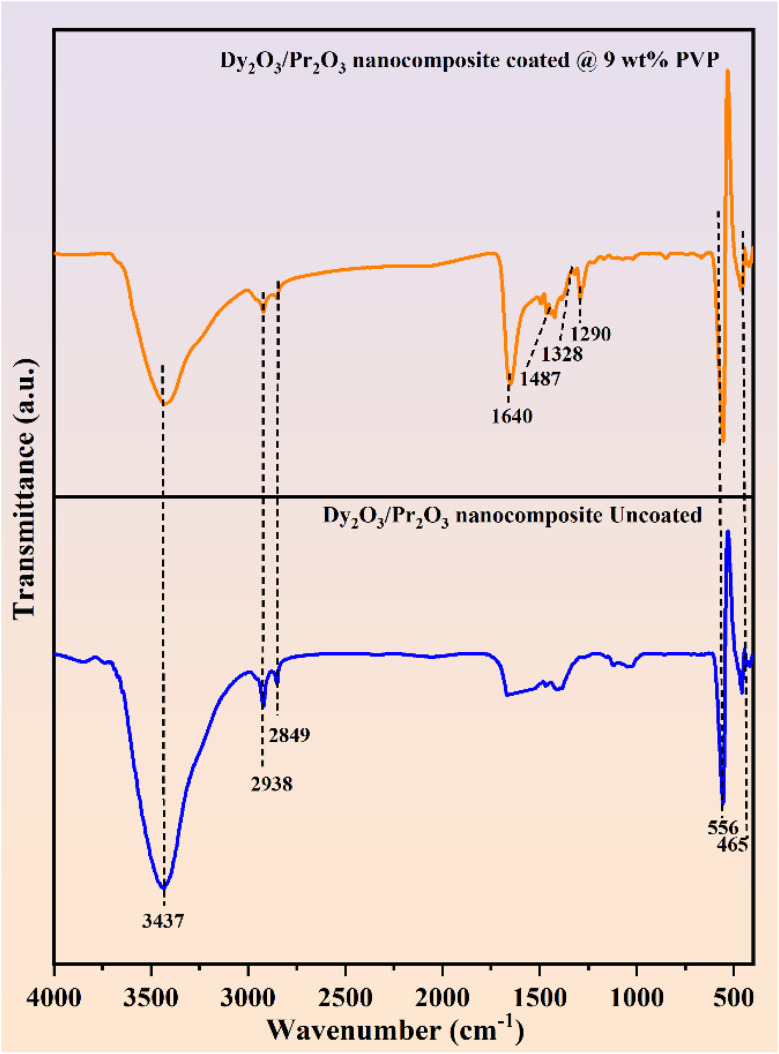
FTIR spectra of uncoated and 9 wt% PVP coated Dy_2_O_3_/Pr_2_O_3_ nanocomposite.

FTIR spectra of the uncoated and 9 wt% PVP-coated Dy_2_O_3_/Pr_2_O_3_ nanocomposites are shown in Fig. 2. The uncoated sample exhibits characteristic metal–oxygen (M–O) stretching vibrations at 556 cm^−1^ and 465 cm^−1^, corresponding to Dy–O and Pr–O bonds, respectively. A broad band at 3437 cm^−1^ is attributed to O–H stretching due to surface water absorption, while peaks at 2938 cm^−1^ and 2849 cm^−1^ arise from residual C–H stretching, possibly from combustion by-products. After PVP coating, new peaks appear at 1640 cm^−1^, 1487 cm^−1^, 1328 cm^−1^, and 1290 cm^−1^, corresponding to the C=O stretching, C–N stretching, and CH_2_ bending modes of the PVP polymer are observed. The reduced intensity of the O–H band indicates effective surface coverage. The retention of M–O bands confirms that the oxide structure remains intact. These results confirm successful PVP surface functionalization, which enhances dispersion, reduces surface hydroxyl groups, and preserves the core crystalline structure-beneficial for improving optical and application performance.^46,47^

**Fig. 3:**
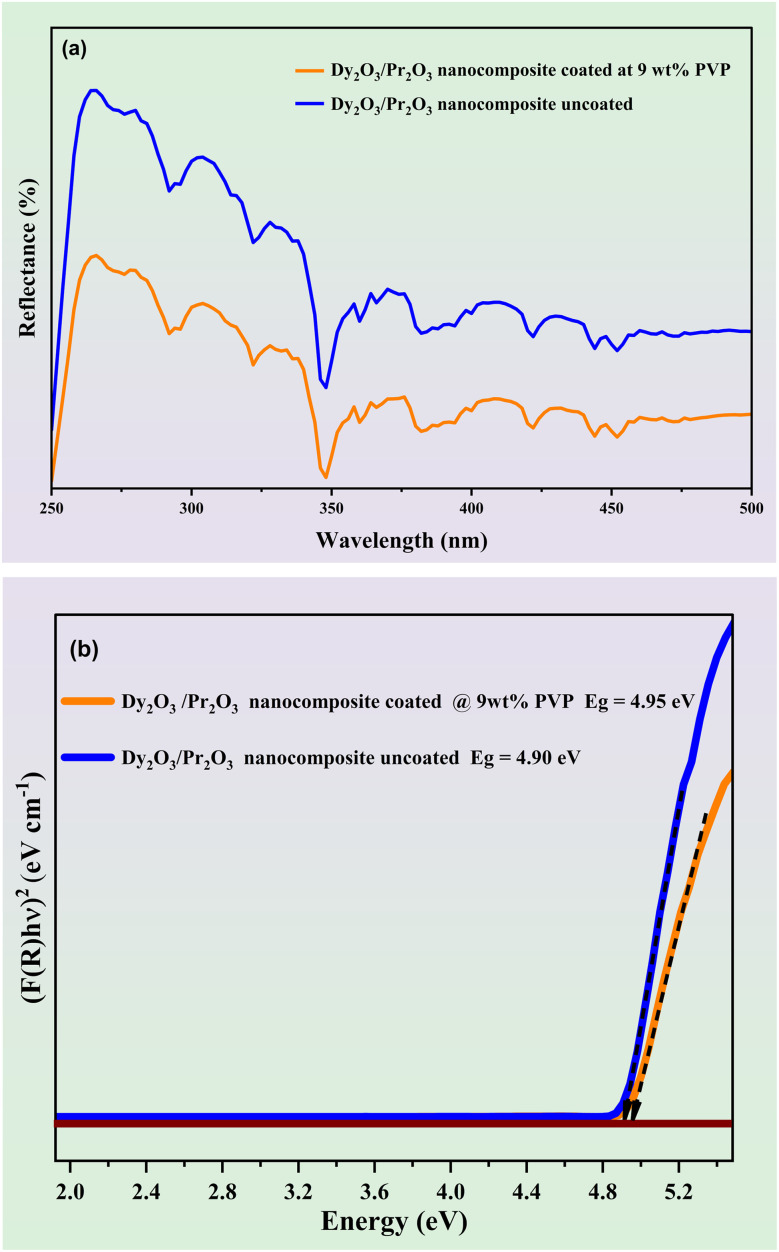
(a) UV-Vis diffuse reflectance spectra and (b) band-gap values of uncoated and 9 wt% PVP coated Dy_2_O_3_/Pr_2_O_3_ nanocomposite.

The optical absorption behavior and band gap energies of the Dy_2_O_3_/Pr_2_O_3_ nanocomposites were evaluated using UV-Vis diffuse reflectance spectroscopy (DRS), as shown in Fig. 3(a and b). Both coated and uncoated samples exhibit broad absorption in the UV region (250–400 nm), attributed to charge transfer transitions and intra-4f electronic transitions of Dy^3+^ and Pr^3+^ ions. The uncoated nanocomposite shows higher reflectance across the entire range compared to the PVP-coated counterpart, indicating reduced light scattering and enhanced absorption in the coated sample due to improved dispersion and surface modification.

The band gap energies (*E*_g_) were calculated from the Kubelka–Munk-transformed,^48^ DRS data using Tauc plots^49^ for direct allowed transitions, as illustrated in Fig. 3(b). The extrapolation of the linear region to the energy axis yielded band gap values of 4.90 eV for the uncoated and 4.95 eV for the PVP-coated nanocomposites respectively and the calculated band gap values of the samples are listed in Table 1. The slight increase in band gap upon PVP coating can be attributed to the reduction in particle size and quantum confinement effects, consistent with XRD results. Additionally, the surface passivation by PVP reduce sub-bandgap defect states, leading to improved optical transparency.

### 3.2. Morphology analysis

**Fig. 4:**
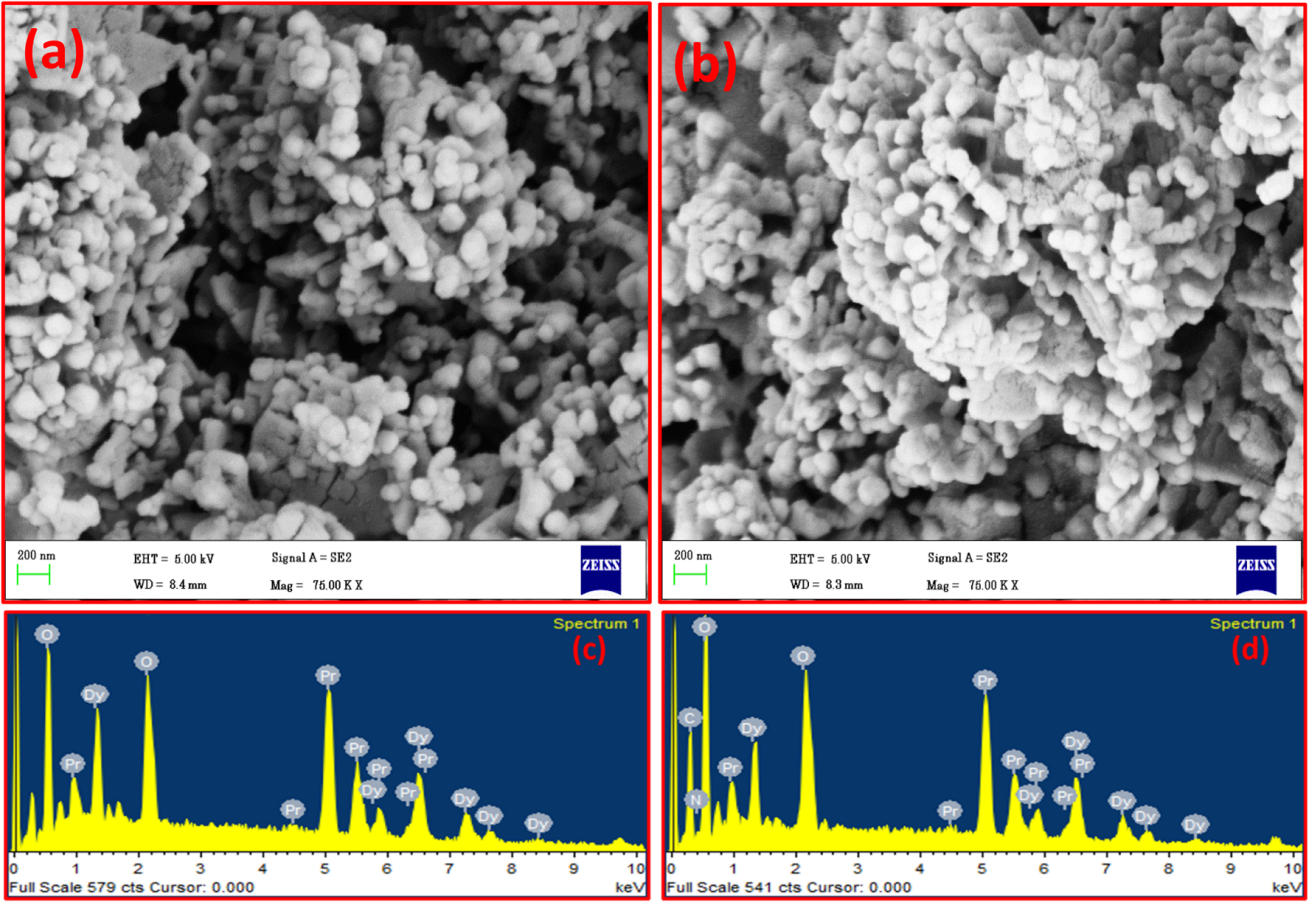
FESEM images of (a) uncoated, (b) coated with 9 wt% PVP; and EDX spectra: (c) uncoated, (d) coated with 9 wt% PVP Dy_2_O_3_/Pr_2_O_3_ nanocomposites.

The surface morphology of the synthesized Dy_2_O_3_/Pr_2_O_3_ nanocomposites were investigated using field emission scanning electron microscopy (FESEM), as shown in Fig. 4(a) and (b). The uncoated sample (Fig. 4(a)) exhibits an aggregated structure composed of irregularly shaped coral like structure forming dense clusters. The particles appear loosely packed with visible intergranular voids, likely due to uncontrolled grain growth and interparticle fusion during high-temperature calcination. The observed morphology is consistent with typical oxide powders prepared *via* solution combustion synthesis. Whereas the PVP-coated sample (Fig. 4(b)) displays a more refined morphology, with relatively uniform particle dispersion and reduced agglomeration. The nanoparticles appear to be embedded in a loosely networked matrix, attributed to the presence of polyvinylpyrrolidone on the surface. The coating likely provides steric stabilization, suppressing nanoparticle coalescence and leading to a more discrete particle distribution. This morphological shift is also consistent with the reduced crystallite size (∼16 nm) observed in XRD, confirming that PVP plays a significant role in modulating particle growth and surface uniformity.

To confirm the elemental composition and distribution of the ions, energy-dispersive X-ray spectroscopy (EDX) was performed, and the recorded spectra is shown in Fig. 4(c) for the uncoated and Fig. 4(d) for the coated samples. In both cases, prominent peaks corresponding to dysprosium (Dy), praseodymium (Pr), and oxygen (O) are observed, confirming the successful formation of the Dy_2_O_3_/Pr_2_O_3_ composite while the elemental composition of uncoated and coated samples listed in Table 2. The relative peak intensities are consistent with the expected stoichiometry. In the coated sample (Fig. 4(d)), a small additional peak corresponding to carbon (C) is detected, which arises from the organic backbone of PVP. The presence of nitrogen (N) is also attributed to the amide group in the pyrrolidone ring. These signals provide direct evidence of successful surface functionalization by the polymer. No impurity elements are detected in either sample, confirming the chemical purity and compositional stability of the synthesized materials.

**Table 2:** Elemental composition of values of uncoated and 9 wt% PVP coated Dy_2_O_3_/Pr_2_O_3_ nanocomposite

Samples	Elements	Weight%	Atomic%
Dy_2_O_3_/Pr_2_O_3_ nanocomposite	O K	12.76	57.90
	Dy L	45.50	23.45
	Pr L	41.74	18.65
Dy_2_O_3_/Pr_2_O_3_ nanocomposite coated with 9 wt% PVP	C K	11.07	38.46
	N K	1.53	4.57
	O K	14.03	36.59
	Dy L	39.32	11.64
	Pr L	34.04	8.74

## 4. Analysis of luminescence properties

**Fig. 5:**
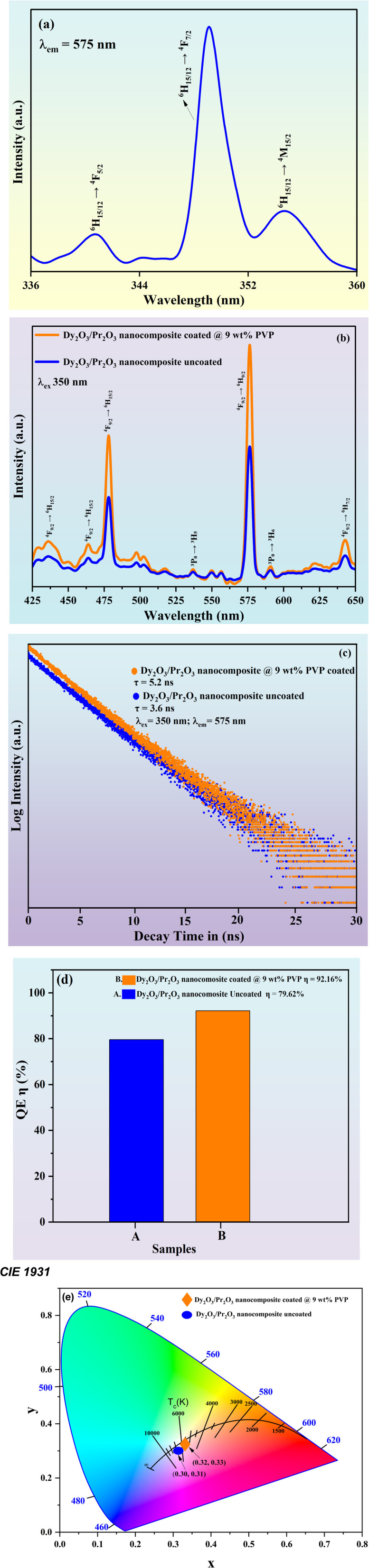
(a) Excitation and (b) emission spectra of Dy_2_O_3_/Pr_2_O_3_ nanocomposite coated with 9 wt% PVP, (c) photoluminescence (PL) decay curves of Dy_2_O_3_/Pr_2_O_3_ nanocomposite coated with 9 wt% PVP and (d) photoluminescence (PL) variation in QE (*η*) of Dy_2_O_3_/Pr_2_O_3_ nanocomposite coated with 9 wt% PVP and (e) CIE & CCT diagram of Dy_2_O_3_/Pr_2_O_3_ nanocomposite coated with 9 wt% PVP.

Fig. 5(a) shows the photoluminescence excitation spectrum of the Dy_2_O_3_/Pr_2_O_3_ nanocomposite, monitored at an emission wavelength of 575 nm, corresponding to the most intense ^4^F_9/2_ → ^6^H_13/2_ transition of Dy^3+^ ions. Several well-resolved excitation bands are evident in the 336–360 nm range, arising from characteristic 4f–4f transitions of Dy^3+^ and Pr^3+^ ions. The peaks observed near ∼343 nm and ∼348 nm are attributed to the ^6^H_15/2_ → ^4^F_5/2_ and ^4^F_7/2_ transitions respectively, while the band at ∼353 nm corresponds to the ^6^H_15/2_ → ^4^M_15/2_ transition. The dominant excitation peak around 350 nm reflects the efficient absorption of UV light through the Dy^3+ 6^H_15/2_ → ^4^F_7/2_ transition, which serves as the primary energy input level for subsequent emission. The sharpness and intensity of these f–f excitation bands confirm the high phase purity and well-preserved crystal field environment within the nanocomposite, with minimal lattice distortion or inhomogeneity.

The photoluminescence (PL) emission spectrum shown in Fig. 5(b) illustrates the characteristic f–f transitions of both Dy^3+^ and Pr^3+^ ions within the Dy_2_O_3_/Pr_2_O_3_ nanocomposite, excited at 350 nm. The most intense peak observed at ∼575 nm corresponds to the electric dipole-allowed ^4^F_9/2_ → ^6^H_13/2_ transition of Dy^3+^, which lies in the yellow region of the visible spectrum and is hypersensitive to the local crystal field environment. This transition dominates the emission profile and is particularly useful for white light generation when combined with blue and red components. The peak at ∼483 nm is assigned to the ^4^F_9/2_ → ^6^H_15/2_ magnetic dipole transition of Dy^3+^, appearing in the blue region and contributing to the spectral balance necessary for warm white light.

In addition to these Dy^3+^ emissions, the spectrum reveals smaller but identifiable features that is attributed to the presence of Pr^3+^ ions. Notably, weak shoulders around ∼530–540 nm and ∼610 nm correspond to transitions involving Pr^3+^ energy levels. The emissions at ∼540 nm and ∼610 nm are attributed to the ^3^P_0_ → ^3^H_5_ and ^3^P_0_ → ^3^H_6_ transitions of Pr^3+^, respectively. These Pr^3+^ transitions, although spin- and parity-forbidden, can be observed in nanostructured rare-earth oxides due to the breakdown of strict selection rules in distorted or asymmetric lattice environments. Their presence adds spectral richness and enhances the chromatic versatility of the nanocomposite. A very weak emission is also observed at ∼650 nm, corresponding to the ^4^F_9/2_ → ^6^H_11/2_ transition of Dy^3+^, which lies in the red spectral region.

The incorporation of Pr^3+^ alongside Dy^3+^ not only introduces additional emission channels but also promotes potential energy transfer pathways, which can broaden the emission profile and fine-tune the color temperature. The enhancement of all emission peaks in the PVP-coated sample, as seen in the increased intensity of both Dy^3+^ and Pr^3+^ transitions, underscores the beneficial role of surface passivation in reducing non-radiative recombination and improving efficiency.

Fig. 5(c) shows the photoluminescence (PL) decay curves of uncoated and 9 wt% PVP coated Dy_2_O_3_/Pr_2_O_3_ nanocomposite were investigated under excitation at 350 nm and emission at 575 nm, the average lifetime (*τ*) was calculated by fitting the decay curves to a single-exponential model.^50^

2 *I*(*t*) = *I*_0_*e*^−*t*/*τ*^

where *I*(*t*) is the emission intensity at time *t*, *I*_0_ is the initial intensity, and *τ* is the decay lifetime. The average lifetime (*τ*) for the uncoated nanocomposite is measured to be 3.6 ns, whereas the PVP-coated nanocomposite displays a significantly longer lifetime of 5.2 ns. This increase in lifetime upon PVP functionalization indicates a reduction in non-radiative recombination processes. The surface capping effect of PVP suppresses surface defects and oxygen vacancies that typically act as quenching centers, thereby enhancing the radiative relaxation pathways of Dy^3+^ ions. Moreover, the extended lifetime supports the enhanced steady-state PL intensity observed earlier, confirming that PVP not only improves emission intensity but also prolongs the excited-state duration, making the coated nanocomposite more suitable for applications requiring sustained luminescent output, such as WLEDs and optical tagging. Importantly, the longer decay lifetime has direct functional implications, in WLEDs, it ensures spectral stability and mitigates transient quenching under continuous electrical pumping, while in optical tagging and anti-counterfeiting, it provides temporally persistent luminescence that remains discernible even under intermittent or weak excitation. Thus, the combination of increased lifetime and higher quantum yield in the coated system underscores its robustness and reliability as a multifunctional optical material.

Fig. 5(d) Shows the QE (*η*) of uncoated and 9 wt% PVP coated Dy_2_O_3_/Pr_2_O_3_ nanocomposite, the quantum yield (QY) of uncoated and 9 wt% PVP coated Dy_2_O_3_/Pr_2_O_3_ nanocomposite were determined using an integrating sphere setup attached to a spectrophotometer, which enabled accurate measurement of absorbed and emitted photon intensities. The quantum yield (*Φ*) was calculated using the formula:3
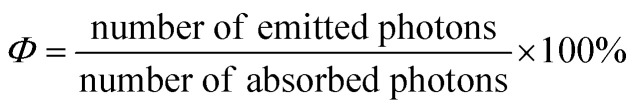


This method involves measuring the total emission spectrum of the sample and the decrease in excitation light intensity due to absorption by the sample inside the integrating sphere.^51,52^ The QE of the coated sample reaches 92.16%, notably higher than the 79.62% observed for the uncoated counterpart. The marked improvement in QE upon PVP functionalization is attributed to effective surface passivation, which reduces non-radiative recombination pathways commonly associated with surface defects and oxygen vacancies. The polymer coating not only minimizes energy loss but also promotes better dispersion of nanoparticles, enhancing excitation efficiency. This result is consistent with the enhanced PL intensity and prolonged decay lifetime observed in previous measurements, affirming that PVP coating substantially improves the radiative efficiency and optical performance of the Dy_2_O_3_/Pr_2_O_3_ nanocomposite.

Fig. 5(e) shows the optical properties of the prepared uncoated and 9 wt% PVP-coated Dy_2_O_3_/Pr_2_O_3_ nanocomposite evaluated using the Commission Internationale de l'Éclairage (CIE) 1931 chromaticity diagram,^53^ and the correlated color temperature (CCT). The chromaticity coordinates (*x*, *y*) of the samples were calculated from photoluminescence data and plotted on the CIE diagram to visualize their emission characteristics. The CCT values were calculated using McCamy's approximation formula, providing insight into the perceived color temperature of the emitted light. The uncoated nanocomposite (blue circle) is located closer to the neutral white emission, with coordinates near the Planckian locus, indicating a CCT of 6132 K and a CRI of 89, suggestive of slightly bluish-white light. In contrast, the PVP-coated sample (orange diamond) shifts toward a warmer white region on the diagram, with a CCT of 5826 K and a CRI of 92, indicating a red-shifted chromatic output and a more balanced emission profile across the visible spectrum. These values, including chromaticity coordinates, CCT, and CRI, were depicted and tabulated in the Table 3.

**Table 3:** Photometric values of Dy_2_O_3_/Pr_2_O_3_ nanocomposite and Dy_2_O_3_/Pr_2_O_3_ nanocomposite coated with 9 wt% PVP

Sample	*x*	*y*	CCT (K)	CRI
Dy_2_O_3_/Pr_2_O_3_ nanocomposite	0.307754	0.317911	6132	89
Dy_2_O_3_/Pr_2_O_3_ nanocomposite coated with 9 wt% PVP	0.327749	0.33692	5826	92

This shift is attributed to the enhancement in yellow and red emission bands upon surface passivation, which increases the contribution from electric dipole transitions of Dy^3+^ (particularly the ^4^F_9/2_ → ^6^H_13/2_ transition) and minor Pr^3+^ emissions. The slightly lower CCT and higher CRI in the coated sample result in emission that is closer to ideal white-light illumination, offering improved visual comfort and more accurate color rendering. Such tuning in chromaticity and CCT is essential for tailoring materials for white light-emitting diode (WLED) applications, where both spectral quality and visual perception are critical. These findings confirm that PVP coating not only enhances the optical efficiency but also enables precise spectral modulation of the Dy_2_O_3_/Pr_2_O_3_ nanocomposite, facilitating its integration into advanced lighting technologies requiring high quantum efficiency, color fidelity, and tunable white-light output.

## 5. Analysis latent fingerprints

**Fig. 6:**
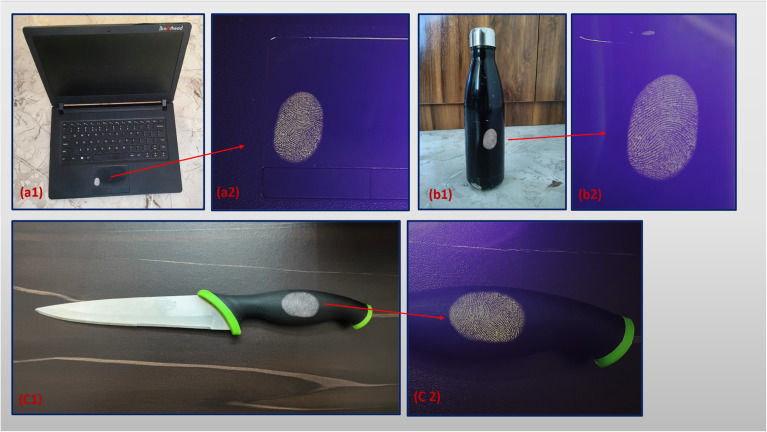
LFPs visualized by powder dusting method by Dy_2_O_3_/Pr_2_O_3_ nanocomposite coated with 9 wt% PVP on the multiple surfaces (a1–c1) under day light and (a2–c2) under 365 nm UV light.

Latent fingerprint (LFP) detection remains a cornerstone of forensic science, serving as a reliable and non-invasive method for personal identification in both criminal and civil investigations.^54,55^ In this study, 9 wt% PVP-coated Dy_2_O_3_/Pr_2_O_3_ nanocomposite powders were employed as a luminescent fingerprint development material to visualize latent fingerprints deposited on various real-life non-porous substrates. The nanocomposite formulation was specifically engineered to combine the sharp 4f–4f transitions of Dy^3+^ and Pr^3+^ ions with the dispersibility and residue affinity imparted by the PVP surface coating. Fingerprints were deposited by direct fingertip contact on challenging surfaces including a black laptop body, a curved stainless-steel bottle, and a polymer knife handle. The powder was applied using the conventional dusting method with a soft forensic brush. It is important to note that the powder was never applied directly to the fingertip; instead, natural fingerprint residues were first deposited on the object surface and subsequently developed by gentle dusting, in accordance with standard forensic practice imaging was conducted under both ambient daylight and 365 nm UV light, as shown in Fig. 6(a1–c2).

Under visible light (Fig. 6(a1–c1)), the fingerprint patterns remained partially obscured due to the dark color and reflective nature of the substrates, which limited ridge contrast. However, under 365 nm UV excitation (Fig. 6(a2–c2)), the nanocomposite emitted strong white-yellow photoluminescence, dramatically enhancing ridge visibility and providing high-fidelity visualization of the entire fingerprint structure. This emission arises from characteristic Dy^3+^ transitions (^4^F_9/2_ → ^6^H_15/2_ and ^4^F_9/2_ → ^6^H_13/2_), supplemented by spectral contributions from Pr^3+^ ions that extend the emission into the orange-red region, improving chromatic balance and visibility under UV illumination. The presence of PVP in the formulation significantly improves nanoparticle interaction with the fingerprint residue. Its hydrophilic functional groups such as carbonyl and pyrrolidone moieties form hydrogen bonds and van der Waals interactions with skin secretions, ensuring preferential binding of the luminescent particles to the ridges rather than to the surrounding substrate.

**Fig. 7:**
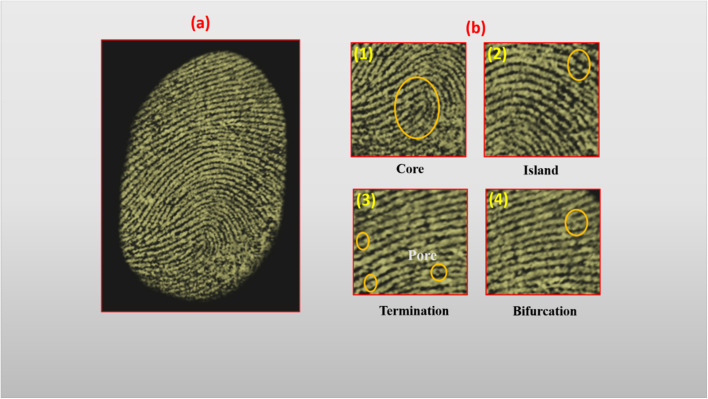
(a) Visualization of latent fingerprint ridge details using Dy_2_O_3_/Pr_2_O_3_ nanocomposite coated with 9 wt% PVP on metal surface under 365 nm UV light and (b) magnified image.

As depicted in Fig. 7(a and b), the developed fingerprints displayed well-defined level 1 ridge features including overall patterns and ridge flow and level 2 minutiae, such as bifurcations, ridge endings, and islands. High-magnification examination of Fig. 7(b1–b4) revealed further evidence of level 3 features, including sweat pores and ridge contour irregularities, which are seldom resolved using traditional fingerprint powders. Specifically, bifurcation points (Fig. 7(b1)), ridge islands (Fig. 7(b2)), the core curvature region (Fig. 7(b3)), and ridge endings with visible pore structures (Fig. 7(b4)) were clearly visualized, affirming the nanocomposite's exceptional spatial resolution. The strong photoluminescent output under low-power UV also ensured minimal background interference and high signal-to-noise ratio, facilitating detailed biometric feature extraction even on reflective or curved surfaces.

The forensic efficiency of this nanocomposite is attributed to a combination of structural and optical mechanisms (i) enhanced luminescence due to efficient Dy^3+^–Pr^3+^ transitions, (ii) surface passivation and dispersion provided by PVP that increases fingerprint residue adhesion, and (iii) excellent photostability, which preserves emission integrity under prolonged UV exposure. Together, these attributes enable high-resolution, multi-level fingerprint visualization on real-world substrates. The adaptability of the powder dusting method, coupled with the nanocomposite's intense UV-induced luminescence, renders it a powerful forensic tool for latent fingerprint detection in field and laboratory settings. This material demonstrates strong potential for integration into practical forensic protocols, especially in scenarios requiring high-resolution, non-destructive, and spectrally tunable fingerprint imaging.

## 6. Analysis of anti-counterfeiting

**Fig. 8:**
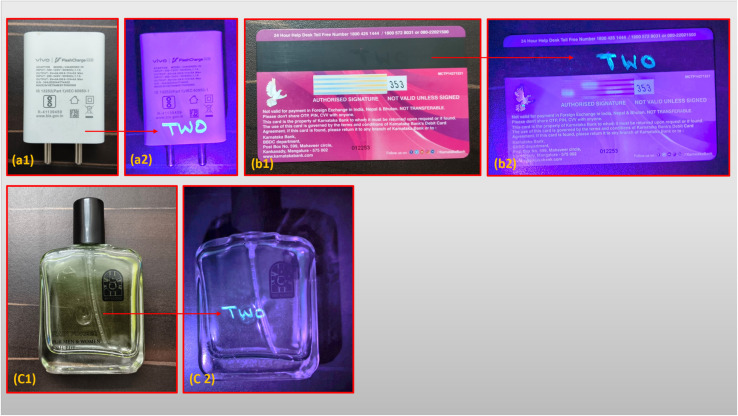
Anti-counterfeiting labels painted with Dy_2_O_3_/Pr_2_O_3_ nanocomposite coated with 9 wt% PVP ink under visible light (a1–c1) and (a2–c2) 365 nm UV light.

In the face of rising global threats from counterfeiting across sectors such as finance, identity verification, and brand protection, the development of smart, covert, and stable optical security materials has become critical.^56^Fig. 8 demonstrates the successful integration of the 9 wt% PVP-coated Dy_2_O_3_/Pr_2_O_3_ nanocomposite as a high-performance anti-counterfeiting agent. The alphanumeric code “TWO” was manually inscribed using a dip-pen writing method on various real-world substrates, a mobile charger (Fig. 8(a1 and a2)), a plastic debit card (Fig. 8(b1 and b2)), and a transparent perfume bottle (Fig. 8(c1 and c2)). Under ambient lighting conditions, the inscribed code remains optically undetectable, offering a covert tagging strategy that avoids visual disruption or aesthetic alteration. However, upon irradiation with a 365 nm UV source, a strong and clearly defined cyan-blue yellow luminescence is observed, resulting from the characteristic 4f–4f transitions of Dy^3+^ ions, primarily the ^4^F_9/2_ → ^6^H_15/2_ transition, subtly enhanced by Pr^3+^ contributions.

The PVP coating plays a crucial role in optimizing ink performance by improving nanoparticle dispersion, suppressing aggregation, and enhancing adhesion to both hydrophobic and hydrophilic substrates. The coating also minimizes surface defects, reducing non-radiative decay pathways and thereby boosting emission intensity and spectral clarity. The luminescent output remains stable even on challenging substrates like transparent and glossy materials, indicating excellent compatibility and durability. The sharp emission contrast between coated and uncoated regions under UV light enables rapid and unambiguous authentication without the need for specialized readers, supporting practical deployment in field-level verification scenarios. Importantly, the ability to write custom security codes with minimal processing or equipment combined with tunable emission characteristics and excellent photostability makes this nanocomposite ink a scalable, low-cost solution for anti-counterfeiting. Its successful demonstration across a spectrum of materials validates its utility in security printing, brand authentication, electronic packaging, and tamper-evident labeling. These findings position the 9 wt% PVP-coated Dy_2_O_3_/Pr_2_O_3_ nanocomposite as a multifunctional luminescent material for next-generation optical security applications, capable of bridging laboratory performance with real-world applicability.

## 7. Conclusion

PVP-functionalized Dy_2_O_3_/Pr_2_O_3_ nanocomposites synthesized *via* solution combustion exhibited cubic phase purity, reduced crystallite size (22 → 16 nm), and strong PVP–oxide interactions leading to enhanced dispersion and bandgap widening (4.95 eV). The nanocomposites showed intense Dy^3+^/Pr^3+^ emissions with high quantum efficiency (92.16%), extended decay lifetime (5.2 ns), and warm white light emission (CCT: 5826 K, CRI: 92), making them promising for WLEDs. Beyond lighting, they enabled high-resolution latent fingerprint visualization under UV light and functioned as invisible luminescent ink for anti-counterfeiting. These findings demonstrate a multifunctional materials platform bridging solid-state lighting, forensic imaging, and optical security.

## Conflicts of interest

There are no conflicts to declare.

## Data Availability

All data generated and analyzed during this study are included in research article in the form of plots/graphs and tables. No additional raw data are available.
